# Behavior of the Edible Seaweed *Sargassum fusiforme* to Copper Pollution: Short-Term Acclimation and Long-Term Adaptation

**DOI:** 10.1371/journal.pone.0101960

**Published:** 2014-07-15

**Authors:** Hui-Xi Zou, Qiu-Ying Pang, Li-Dong Lin, Ai-Qin Zhang, Nan Li, Yan-Qing Lin, Lu-Min Li, Qin-Qin Wu, Xiu-Feng Yan

**Affiliations:** 1 Zhejiang Provincial Key Lab for Subtropical Water Environment and Marine Biological Resources Protection, College of Life and Environmental Science, Wenzhou University, Wenzhou, People's Republic of China; 2 Key Laboratory of Saline-Alkali Vegetation Ecology Restoration in Oil Field, Northeast Forest University, Harbin, People's Republic of China; CINVESTAV-IPN, Mexico

## Abstract

Aquatic agriculture in heavy-metal-polluted coastal areas faces major problems due to heavy metal transfer into aquatic organisms, leading to various unexpected changes in nutrition and primary and/or secondary metabolism. In the present study, the dual role of heavy metal copper (Cu) played in the metabolism of photosynthetic organism, the edible seaweed *Sargassum fusiforme*, was evaluated by characterization of biochemical and metabolic responses using both ^1^H NMR and GC-MS techniques under acute (47 µM, 1 day) and chronic stress (8 µM, 7 days). Consequently, photosynthesis may be seriously inhibited by acute Cu exposure, resulting in decreasing levels of carbohydrates, e.g., mannitol, the main products of photosynthesis. Ascorbate may play important roles in the antioxidant system, whose content was much more seriously decreased under acute than that under chronic Cu stress. Overall, these results showed differential toxicological responses on metabolite profiles of *S. fusiforme* subjected to acute and chronic Cu exposures that allowed assessment of impact of Cu on marine organisms.

## Introduction

Over the last few decades, heavy metal pollution has become a global problem posing threat on both soil and marine ecosystems, as a result of the mass industrialization and various agricultural activities such as the intensive use of chemical fertilizers, wastewater and biosolids [1]. Many heavy metals accumulate in marine organisms, which may be subsequently transferred to human body via the food chain [2].

Marine algae, particularly seaweeds, are a food source for marine animals such as sea urchins and fishes, and are the base of many marine food webs. For several centuries, there has been a traditional use of seaweeds as food in East-Asian countries, like China, Japan and the Republic of Korea. *Sargassum fusiforme* (Sargassaceae, Phaephyceae), an endemic brown algae from the western coast of the North Pacific, is widely consumed in Japan and Korea. This alga, in great demand, is also cultivated in East-Asian counties, especially in China, where the cultivation area was 2.6% (2,482 ha) of the entire coastal area for commercial cultivation of seaweeds with a total production reached 32,000 tonnes per year (freshweight) [3].

It is reported that concentration of copper (Cu) found in standard reference material (oyster tissue) from China was above 100 µg g^−1^ [4], indicating a serious Cu pollution in these coastal areas. Although Cu is an essential micronutrient, excessive amount can be extremely harmful to algae [5]. Seaweeds are often exposed to low concentrations of metals including Cu for long periods. In the cases of ocean outfall, they may even abruptly exposed to high levels of metals. In the study of short- and long-term response of the marine green macroalga *Ulva fasciata* to Cu excess, regulation of mRNA expression involved in redox homeostasis and antioxidant defense were different [6]. In another study, distinct changes in the antioxidant responses to acute or chronic treatment with Cu were observed in the unicellular alga *Gonyaulax polyedra*, suggesting a different oxidative status of these two types of metal stresses [7]. Thus, it seems that both micro- and macroalgae have different responsive mechanisms to short- and long-term exposures of Cu.

Chemical analysis alone is not able to provide a satisfying assessment of the environmental quality of an ecosystem due to the biotransforming of an individual pollutant by living organisms [8], [9]. To gain more information regarding the health state of a particular ecosystem, it is important to monitor the response of biota to the pollutants as well. Metabolomics characterizes and quantifies end products-the metabolites that produced by living organismsunder a given set of conditions. Metabolomics has shown considerable potential as a tool for environmental toxicology [10]–[14]. Both GC-MS and NMR techniques have been widely used in metabolomics and metabolite profiling [15]–[18]. GC-MS is particularly effective in the analysis of primary metabolites, while NMR, inherently quantitative, provides universal detection for organic components without coupling to a separation technique [19], [20]. Because of the complementary analytical features of NMR and MS, opportunities for leveraging both methods are being considered which will create a more comprehensive metabolic profiling [19], [21]–[22].

It is now well known that synthesis of antioxidant and metal-chelating components and activation of antioxidant enzymes are key factors for tolerance to heavy metals and other abiotic stress in plants [23]. The toxic effect of heavy metals appears to be related to production of reactive oxygen species (ROS), which usually leads to lipid peroxidation and oxidation of some enzymes and a massive protein. To better understand oxidative stress under acute and chronic conditions, the content of malondialdehyde (MDA), which represents the level of lipid peroxidation, was measured, as well as the activities of antioxidant enzymes superoxide dismutase (SOD), catalase (CAT) and peroxidase (POD). Additionally, activity of nitrate reductase (NR) that primarily involved in maintenance of a favorable cellular oxidation/reduction potential was also determined. In this study, we characterized the impact of Cu on the marine brown algae *S. fusiforme* using both NMR- and GC-MS-based metabolomics, which allowed identifying more analytes and created an opportunity to expand the scope of metabolomics research.

## Materials and Methods

### Algal material and culture conditions

*S. fusiforme* samples were collected from the Northeastern coast of Wenzhou, China (28.0°N, 121.2°E) in September 2012. This location is not privately-owned or protected in any way, thus no specific permissions were required, and the field studies did not involve endangered or protected species. After collection, algae were immediately transported to the laboratory in a cooler (4°C) within 2 h. Fronds were then washed with filtered natural seawater (with salinity of 27 ‰) and maintained in high-density polypropylene containers for 2 days at 20°C before Cu treatment, using a photoperiod of 12∶12 h and a photon flux density of 100 µmol m^−2^ s^−1^. Culture medium was aerated and changed daily.

Two experiments were carried out to evaluate the metabolic differences between responses of *S. fusiforme* to acute and chronic Cu exposures. For short-term treatment (1 day), thalli were cultivated in filtered natural seawater containing CuCl_2_ (Sigma, USA) in the final concentration of 47 µM. For long-term treatment (7 days), culture medium containing CuCl_2_ was prepared in the same manner as for short-term treatment but with final concentration of 8 µM. After treatment, thalli were immediately frozen in liquid nitrogen and stored at −80°C for further analysis.

In addition, the term “acute exposure” was employed for conditions of exposure to high Cu concentrations (47 µM) after 1 day in this study. On the other hand, “chronic exposure” defines the exposure to lower sub-lethal Cu concentrations (8 µM) after 7 days.

### Measurement of enzyme activities and MDA content

Approximately 0.1 g algal samples (fresh weight, *n* = 5) were homogenized in liquid nitrogen and extracted with 1 mL of 0.05 M potassium phosphate buffer (pH 7.0) containing 0.25% (v/v) Triton X-100 and 1% (w/v) polyvinylpolypyrrolidone (PVPP). The enzyme activities in the algal tissues were detected using commercial kits (Nanjing Jiancheng Biotech., China) according to the manufacturer's instructions. In this study, the antioxidant enzymes included SOD (EC 1.15.1.1), CAT (EC 1.11.1.6) and POD (EC 1.11.1.7). In addition, activity of nitrate NR (EC 1.7.99.4) was also determined.

Protein concentration was determined according to the method of Bradford [24] with bovine serum albumin as standard. The unit of each enzyme was defined as the activity of an enzyme per milligram of total protein (expressed in µmol min^−1^ per mg protein, or U per mg protein).

As a measure of lipid peroxidation, MDA levels in algal tissue were estimated by measuring thiobarbituric acid reactive substances following the standard protocol using MDA detection kit (Nanjing Jiancheng Biotech., China) and were expressed as nmol per mg of protein.

### NMR analysis and data processing

Polar metabolites were extracted from the algal tissues using the solvent system of methanol/water (1/1) as described previously [25], [26]. NMR spectra were acquired using Bruker AV-500 spectrometer (Bruker Bio Spin, Canada), with ^1^H observation frequencies of 500.18 MHz, spectral width 6,009.6 Hz, mixing time 0.1 s, and relaxation delay 0.3 s as described previously [27].

All the NMR spectra were converted to a format for pattern recognition analysis using custom-written ProMetab software based on the Matlab software package (version 7.0; The MathWorks, Natick, MA, USA) [28]. NMR spectral peaks were identified following tabulated chemical shifts [29] and using the software, Chenomx (Evaluation Version, Chenomx Inc., Canada).

### GC-TOF MS analysis and data processing

Extraction and fractionation of metabolites for GC-TOF MS analysis were performed as described [30] and about 100 mg of each tissue sample was weighed accurately. After derivatization, the metabolites were analyzed by GC-TOF MS analysis that was performed using an Agilent 7890 gas chromatograph system (Agilent, CA, USA) coupled with a Pegasus 4D time-of-flight mass spectrometer (LECO Corp., MI, USA). The system utilized a DB-5MS capillary column coated with 5% diphenyl cross-linked with 95% dimethylpolysiloxane (30 m×250 µm inner diameter, 0.25-µm film thickness; J&W Scientific, Folsom, CA, USA). A 1 µL aliquot of the analyte was injected in splitless mode. Helium was used as the carrier gas, the front inlet purge flow was 15 mL min^−1^, and the gas flow rate through the column was 1 mL min^−1^. The initial temperature was kept at 80°C for 0.2 min, then raised to 190°C at a rate of 10°C min^−1^, then to 220°C at a rate of 3°C min^−1^ and finally to 280°C at a rate of 20°C min^−1^ for 16.8 min. The injection, transfer line, and ion source temperatures were 280, 270, and 220°C, respectively. The energy was −70 eV in electron impact mode. The mass spectrometry data were acquired in full-scan mode with the m/z range of 20–600 at a rate of 10 spectra per second after a solvent delay of 480 s.

A total of 288 peaks were found in the 24 samples. Data processing was performed as following: after the missing values were imputed using *k* nearest neighbor method of Bioconductor (www.bioconductor.org) impute package, data was filtered in order to eliminate the noise using interquantile range, resulting in 268 peaks. The filtered data were subsequently normalized where adonitol, pentakis (trimethylsilyl) ether were used as the internal references.

### Statistical analysis

Data were expressed as the mean ± standard deviation (SD). Statistical analysis included one-way analysis of variance (ANOVA). Principal component analysis (PCA) was used to reduce the dimensionality of data and summarize the similarities and differences between multiple NMR spectra. The principal component score plots were used to visualize general clusters between various groups of samples. ANOVA was conducted on PC scores from each group to test statistical significance (*P*<0.05) of separations.

## Results and Discussion

**Figure 1:**
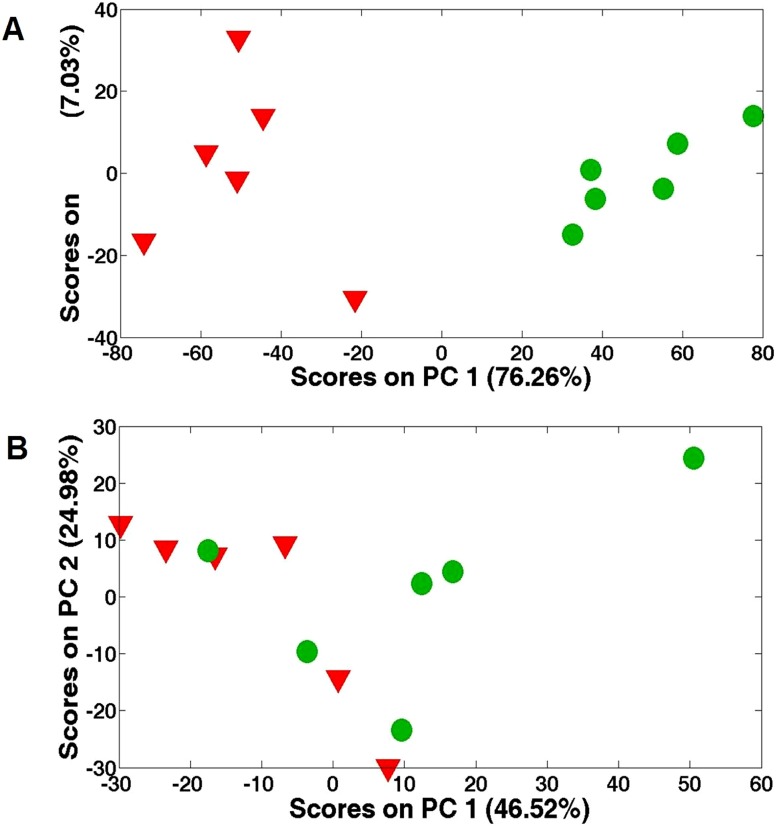
PCA score plots. (A) from the analysis of the 1D ^1^H NMR spectra of *Sargassum fusiforme* tissue extracts from high concentration copper-treated (47 µM Cu^2+^) group after exposure for 1 day; (B) from the analysis of the 1D ^1^H NMR spectra of *Sargassum fusiforme* tissue extracts from low concentration copper-treated (8 µM Cu^2+^) group after exposure for 7 days.

In total, 25 metabolites were exclusively quantified by NMR (Table 1 and 2), while this number for MS was much more (288 peaks detected). Various metabolite classes were identified in NMR spectra, including amino acids, carbohydrates, and intermediates in the tricarboxylic acid (TCA) cycle. Fig. 1A shows the score plots for the acute Cu stress experiment, where PC1 and PC2 represent 76.26% and 7.03%, respectively. The acute Cu stress experiment score plots contain six distinct groups that represent the control samples and the individual treatments. The separations between the control (inverted red triangles) and exposed (green cycles) were obviously observed from the PC scores plots (*P*<0.05), while no separation for the chronic stress (Fig. 1B).

**Table 1:** Concentrations of amino acids and related components.

	Acute stress			Chronic stress		
µmol g^−1^	Control	Treated	Percentage	Control	Treated	Percentage
Alanine	0.2743	0.3557±0.0427*	130%±16%	0.2096	0.2978±0.0428*	142%±20%
Glutamate	0.6674	0.4692±0.0735*	70%±11%	0.4509	0.4907±0.0888	109%±20%
Glutamine	0.1216	0.1324±0.0412	109%±34%	0.0177	0.0598±0.0240*	338%±136%
Aspartate	0.8634	0.5878±0.0763*	68%±9%	0.5140	0.6744±0.0851*	131%±17%
Tryptophan	0.0237	0.0335±0.0024*	141%±10%	0.0351	0.0369±0.0079	105%±23%
Tyrosine	0.0382	0.0626±0.0082*	164%±21%	0.0332	0.0310±0.0126	93%±38%
Phenylalanine	0.0556	0.0480±0.0072	86%±13%	0.0835	0.0655±0.0142	78%±17%
Glycine	0.2329	0.1175±0.0380*	50%±16%	0.2029	0.1211±0.0141*	60%±7%
Isoleucine	0.0325	0.0200±0.0073	62%±22%	0.0266	0.0205±0.0023*	77%±9%
Leucine	0.0238	0.0138±0.0048*	58%±20%	0.0068	0.0095±0.0041	140%±60%
Valine	0.0439	0.0458±0.0061	104%±14%	0.0241	0.0212±0.0022*	88%±9%
N,N-Dimethylglycine	0.0444	0.0226±0.0040*	51%±9%	0.0469	0.0080±0.0019*	17%±4%

The absolute concentration of components in the control condition is shown the first column of each treatment, followed by the absolute concentration of each treatment and the relative changes compared to each control. Asterisk indicates significant differences between the stress and its control condition (*P*<0.05).

**Table 2:** Concentrations of organic acids, sugars, polyols and related components.

	Acute stress			Chronic stress		
µmol g^−1^	Control	Treated	Percentage	Control	Treated	Percentage
Mannitol	19.7659	5.4364±1.2094*	28%±6%	19.9864	17.2164±1.1147*	86%±6%
Malate	0.1094	0.0275±0.0105*	25%±10%	0.0434	0.0261±0.0053*	60%±12%
myo-Inositol	0.1238	0.0986±0.0154	80%±12%	0.0803	0.0447±0.0110*	56%±14%
Citrate	0.4010	0.2056±0.0144*	51%±4%	0.2269	0.1915±0.0316	84%±14%
Xylose	0.1538	0.0997±0.0298	65%±19%	0.2121	0.1254±0.0293*	59%±14%
Succinate	0.0571	0.0302±0.0062*	53%±11%	0.0897	0.0520±0.0032*	58%±4%
Ascorbate	0.1087	0.0110±0.0061*	10%±6%	0.0720	0.0300±0.0064*	42%±9%
Lactate	0.3113	0.0652±0.0130*	21%±4%	0.0314	0.0357±0.0038	114%±12%
Trimethylamine N-oxide	0.2342	0.1628±0.0603*	70%±26%	0.2293	0.2147±0.0313	94%±14%
Trimethylamine	0.0070	0.0052±0.0011	74%±16%	0.0030	0.0069±0.0013*	230%±43%
Carnitine	0.0385	0.0297±0.0078*	77%±20%	0.0335	0.0318±0.0049	95%±15%
Acetate	0.0336	0.0215±0.0018*	64%±5%	0.0296	0.0269±0.0037	91%±13%
O-Phosphocholine	0.0347	0.0184±0.0055*	53%±16%	0.0363	0.0324±0.0051	89%±14%

The absolute concentration of components in the control condition is shown the first column of each treatment, followed by the absolute concentration of each treatment and the relative changes compared to each control. Asterisk indicates significant differences between the stress and its control condition (*P*<0.05).

**Figure 2:**
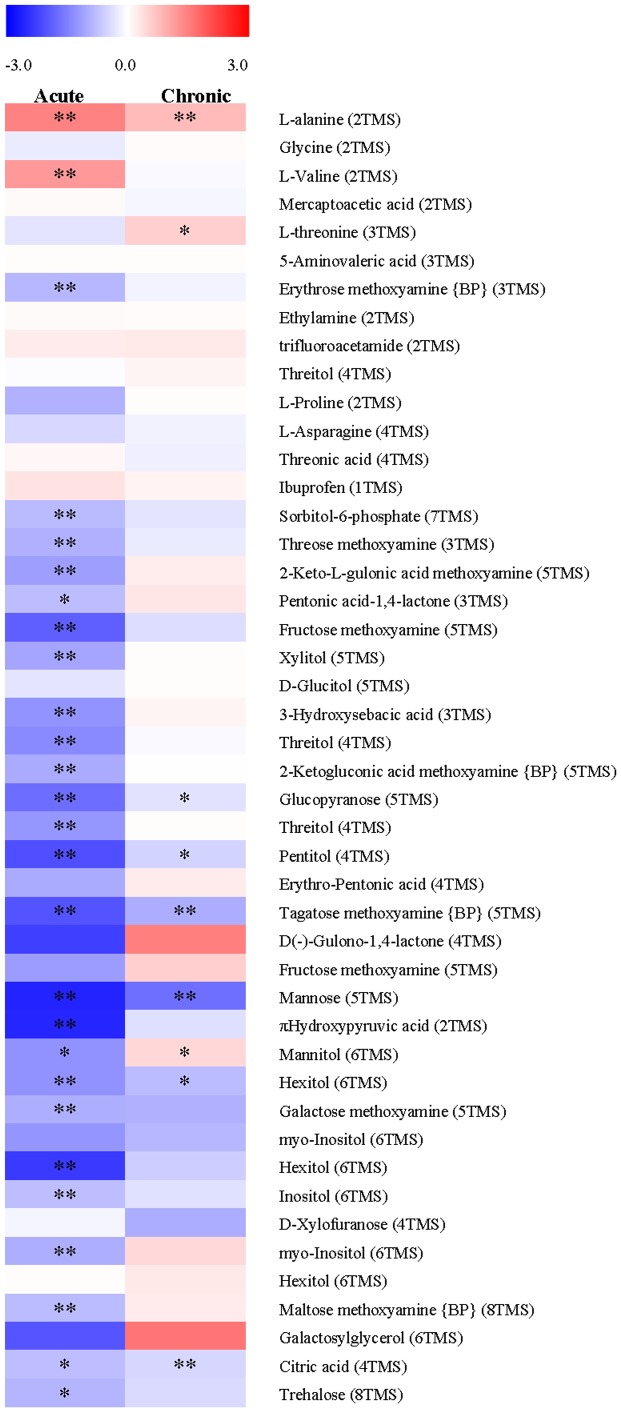
Heat-map of metabolic changes in *Sargassum fusiforme* under acute and chronic Cu stress. Intensity of colors represents log_2_-transformed ratios of measured means (n = 6) analyte's intensity to its respective mean value in the control conditions. Asterisks mark *t*-test *P*-value, where “**” marks *P*<0.01 and “*” marks *P*<0.05.

GC-TOF MS is also a powerful analytical tool employed in metabolomics studies, which provides the detection of different metabolite peaks. The components measured by GC-TOF MS that Fig. 2 summarizes are mainly amino acids, amines, organic acids, polyols and sugars. It has been proposed that decrease in utilization of carbohydrates for growth produced by heavy metals is more pronounced than the decrease in CO_2_ fixation resulting in an increased accumulation of carbohydrates [31]. However, as shown in Fig. 2, the levels of most carbohydrates, e. g., mannose, fructose, hexitol and manitol, were decreased under acute stress, so were those under chronic Cu stress, though to a lesser extent and some even with increasing levels.

### Amino acid concentrations were mainly affected

The summarized results for the amino acids profiling from NMR are shown in Table 1. Generally, more than half of the amino acids exhibit different profiles in response to the acute and chronic Cu stress. Concentrations of alanine and glutamine increased (by 30% and 9%, respectively), while those of aspartate and glutamate significantly decreased (by nearly 30%), resulting in a general decrease in total amount of the predominant amino acids (alanine, glutamate, glutamine and aspartate) under acute stress. It is particularly interesting as all the predominant amino acids content increased, especially glutamine (by 338%), under chronic stress.

**Figure 3:**
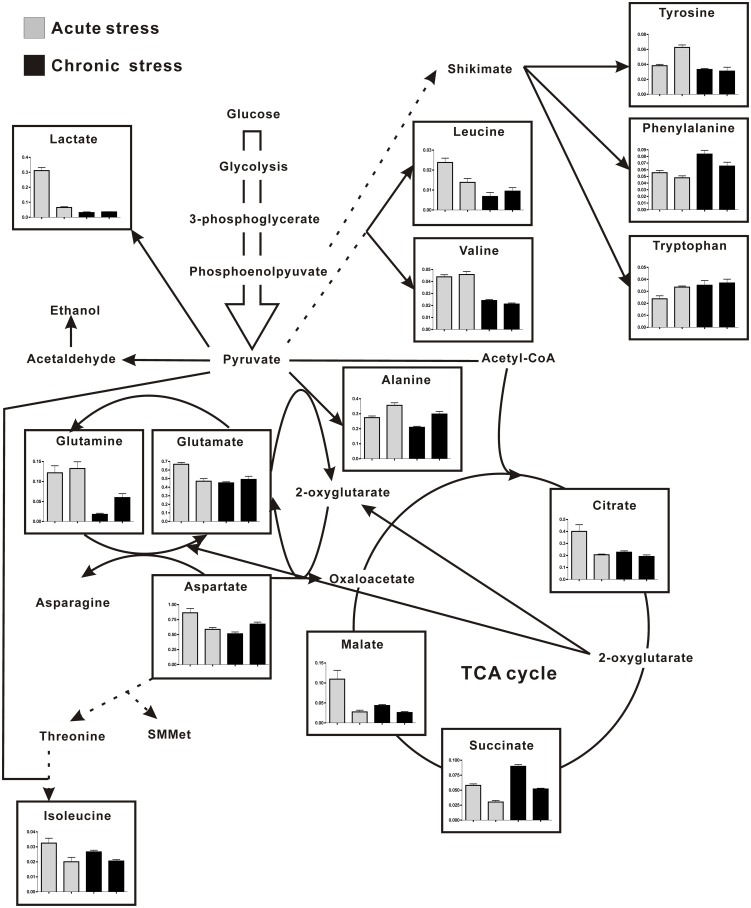
Pathway diagram with bar graphs representing relative metabolite abundance under acute and chronic Cu treatments. The first bar represents the control followed by the Cu treatment of the second bar.

Branched chain and aromatic amino acids were present only in small quantities in *S. fusiforme*. Interestingly, aromatic amino acids content increased (by 23%, Table 1) under acute stress, in contrast, which decreased by 22% under chronic stress. In more detail, the content of tyrosine and tryptophan both increased by approximately 50% except that of phenylalanine, decreased by 14% under acute stress, while minor changes or a slight decrease were observed for aromatic acids under chronic stress, indicating a quite different responding pattern to these two Cu stress in *S. fusiforme*. Aromatic amino acids are synthesized from the common precursor metabolite chorismate, which originates from the shikimate pathway as shown in Fig. 3 [32], whose importance was demonstrated by the fact that 20% of the carbon fixed by plants flows through it under normal growth conditions [33]. Presence of the shikimate pathway in macroalgae has been experimentally verified only in green and red algae, as well as in the diatom *Thalassiosira pseudonana* [34]. However, the reduction in phlorotannin content and mortality in *Fucus vesiculosus* caused by glyphosate indicated the existence of the shikimate pathway in brown algae [35]. Moreover, little is known about the influence of heavy metal stress on enzymes involved in the shikimate pathway [33], especially in algae. Surprisingly, our results indicated an enhancement of the shikimate pathway under acute but not chronic Cu stress. In pepper, due to the much more accumulated Cu in the roots than in the aerial parts, the induction of shikimate dehydrogenase (SKDH), which is the enzyme that catalyses the fourth step in the shikimate pathway, only existed in the hypocotyl [33]. It seems that the amount we used were both not high enough to inhibit the activity of this enzyme. However, influences of different concentrations of Cu on other enzymes involved in the shikimate pathway are also unclear. One hypothesis could be that more other enzymes were induced under acute than chronic Cu stress. Moreover, phenylalanine is required for the synthesis of various phenolic compounds that play important roles in non-enzymatic antioxidant defense processes. This may explain the decrease in the content of phenylalanine under both Cu stress conditions.

### Different nitrogen assimilation patterns

**Figure 4:**
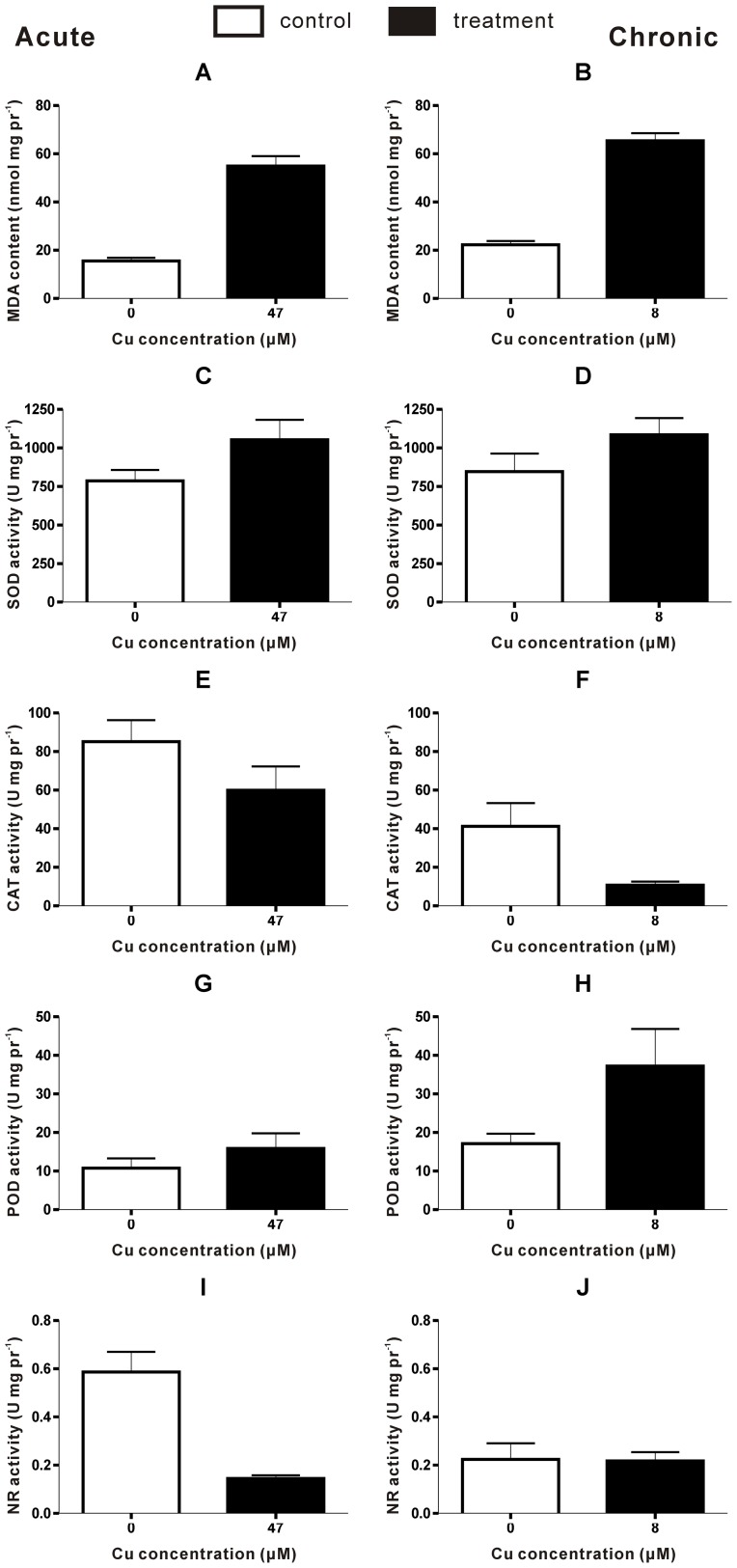
MDA content, antioxidant enzyme activities in *Sargassum fusiforme* under acute and chronic copper stress. Bars represent mean values of independent replicates ±1 SD (n = 4 or 5).

Nitrate assimilation is an apparently simple process in photosynthetic eukaryotes, involving two transports and two reduction steps to produce ammonium in the chloroplast [36], within which NR is a key enzyme that catalyzes the first, also the rate-limiting step in the reduction of nitrate to ammonium [37]. After a short exposure to Cu (1 day), a significant decrease in the NR activation state was observed (Fig. 4I). However, after 7 days of Cu treatment, the NR activation state in thalli was found to be approximate to that in untreated thalli (Fig. 4J). The strong inhibition of NR activity by acute Cu exposure in *S. fusiforme* was entirely in agreement with results obtained with other organisms [38], [39]. Furthermore, excessive Cu causes a drastic change in nitrogen metabolism affecting also other enzymes involved in nitrate reduction and amino acid metabolism and leading to diminution of total nitrogen [40]. As a result, the levels of primary amino acid products of nitrogen assimilation (glutamine and glutamate) were reduced.

During chronic Cu exposure, the total content of glutamate and glutamine, especially glutamine in *S. fusiforme* even increased comparing to the control (Table 1), meanwhile the activity of NR was little reduced (Fig. 4). Though excessive cadmium could significantly inhibit activities of glutamate dehydrogenase (GDH), glutamine synthetase (GS) and glutamine oxoglutarate aminotransferase (GOGAT) [41], through which ammonium is further incorporated into the amino acids [42], [43], data concerning the inhibitory effect of excess Cu are scarce [44]. It is reported that GS appeared highly increased in the model brown alga *Ectocarpus siliculosus* exposed to 50 mg L^−1^ of Cu after 10 days [45]. Consequently, it may suggest that GS be strongly activated in *S. fusiforme*, though whose activity was not determined in the present study, resulting in the dramatic increase in glutamine content. The ratio of glutamine to glutamate has been proposed as an indicator of nitrogen status [46], which was raised in thalli under both acute and chronic Cu stress, by approximately 55% and 200% (Table 1), respectively, showing great variations where the latter was almost 4 times as the former.

### Physiological responses

The algae were cultivated with increasing concentrations of Cu, which would trigger the synthesis of ROS that led to lipids peroxidation [47]. In general, increases in peroxidase activity are regarded as a reliable indicator of stress or potential phytotoxicity of heavy metals, the increase in peroxidase activity being a response to an increase in peroxides [48]. As a product of lipid peroxidation that accumulates greatly following heavy metal exposure, MDA is an indicator of lipid peroxidation. The algae responded similarly in the content of MDA to both acute and chronic Cu stress, resulted in 254% and 193% increase, respectively (Fig. 4A and B).

SOD has been called the cell's first line of defense against ROS that catalyzes the disproportionation of O_2_^−^ to O_2_ and H_2_O_2_ [49]. Generally speaking, peroxidative stress triggers higher level of MDA or lower level of SOD or both. In *S. fusiforme*, SOD activity was activated, a little increased by 34% and 28% (Fig. 4C and D), respectively, under acute and chronic Cu exposure, consistent with the result reported [47], which was also coinciding with increases in MDA content (Fig. 4A and B).

CAT is another important ROS-scavenging enzyme associated with antioxidant stress in algae, which catalyzes the production of H_2_O and O_2_ from the degradation of H_2_O_2_ in cytosol and persoxisomes [50]. It is reported that at high concentrations (above 20 µM), Cu might be responsible for the inhibition of CAT, resulting in an insufficient ROS detoxification with enhanced H_2_O_2_ accumulation and lipid peroxidation [51]. Furthermore, inhibition of CAT activity may be caused by increased levels of O_2_^−^ [52]. However, the activity of CAT declined significantly by 74% in thalli under chronic stress (Fig. 4F), while surprisingly only by 30% in that under acute stress (Fig. 4E). In addition, proper levels of Cu might lead to an increase in activity of CAT to cope with Cu stress [53]. As expected, CAT activity was activated and significantly increased in thalli under 8 µM Cu treatment after 1 day in our study (data not shown). Thus, it is plausible that the only explanation might be that O_2_^−^ accumulated under chronic Cu exposure was more than that under acute treatment. In other word, it is the long-term exposure, rather than the high levels, as the main reason that led to the more accumulated O_2_^−^. In another aspect, the results above also suggested CAT as a more sensitive antioxidant enzyme than SOD, in agreement with other studies [47].

Besides SOD, activity of POD was also up-regulated in our experiment by both treatments, where chronic stress induced more activation of POD activity than acute stress (by 118% and 47%, respectively, Fig. 4H and G).

### Mannitol as the main product of photosynthesis

ROS were formed either after acute or chronic heavy metal exposure, where the former abruptly generated into high levels that exceeded the ability of the antioxidant system to cope with them, while the latter increased steadily, resulting in different levels of damage to cellular compounds [7].

Mannitol is almost universally present in brown algae, being the main product of photosynthesis instead of sucrose [54], which may also function as carbohydrate storage, translocatable assimilate, source of reducing power, osmoregulator and/or antioxidant [55]. Changes in the monnitol content of marine brown algae have been reported in many field-based studies except that of heavy metal [56]. Based on the visual inspection, mannitol is the most abundant metabolite in the NMR spectrum from tissues of *S. fusiforme* (data not shown). The concentration of mannitol decreased strongly by 72% (Table 2) in algae under acute stress, while only by 14% (Table 2) in that under chronic stress.

A mannitol cycle has been proposed in a number of organisms, including micro and macroalgae, where the latter is essentially the same as the fungal cycle [55]. In some yeasts, Cu^2+^ supplementation activates mannitol dehydrogenase involved in the biosynthesis of mannitol, resulting in an increased mannitol production. However, little is known about the affection of Cu on these enzymes involved in the metabolism of mannitol, especially in brown algae.

At the cell membrane, Cu may interfere with cell permeability [57], [58]. In the present study exces Cu treatment caused much mannitol lost in the cell of *S. fusiforme*, indicating an enormous increase in permeability to it. In another aspect, as a compatible solute, mannitol is frequently used as a scavenger of hydroxyl radicals *in vitro* [59] and *in vivo* [60]. It may be involved in the cellular ROS-scavenging system to detoxify the oxidative stress. Therefore, it seems that in the long-term adaptation to low-concentration Cu stress of this algae, no significant differences were observed in the cell permeability, resulting in a little reduction in the content of mannitol.

### Malate and aspartate may play important roles

Similar to C_4_ plants, malate and aspartate were accumulated as candidates for the organic store in *Fucus* spp. that has a quite close phylogenetic relationship with *Sargassum* spp., both of which belong to Fucales [61]. Additionaly, C_3_- and CAM-like photosynthesis were also observed in this species [61]. This coexistence of different photosynthetic pathways may be normal in aquatic environment [62], e.g., the both C_3_ and C_4_ photosynthetic pathways involved in the green-tide-forming alga, *Ulva prolifera* [63]–[65]. Anyway, malate and aspartate may play important roles in photosynthesis. As a potent inhibitor of photosynthesis, Cu dramatically reduced the levels of malate and aspartate in acute Cu treated *S. fusiforme*, where the former was even more strongly reduced (Table 1 and 2). However, content of aspartate was surprisingly increased by nearly 30% when under chronic Cu stress (Table 1). Moreover, malate can function as a vacuolar osmolyte and may also serve as an additional sink for carbon assimilation and reducing equivalents [66].

Aspartate was found to be the most abundant amino acid, which is in line with the results previously described [67]. More than half of the content was D-aspartate, whose cellular localization was also confirmed in *S. fusiforme* [68]. It was proposed that D-aspartate may play an important role in the growth of *S. fusiforme*, as well as in both germination and growth of higher plants [68], [69]. However, the two isomers of aspartate were not elucidated by the methods of our study. To gain deeper insight into the biological role, especially under Cu stress, further studies, for example, the influences of Cu on the levels of both isomers and genes involved in their metabolism, will be required.

### Metabolites involved in choline metabolism

**Figure 5:**
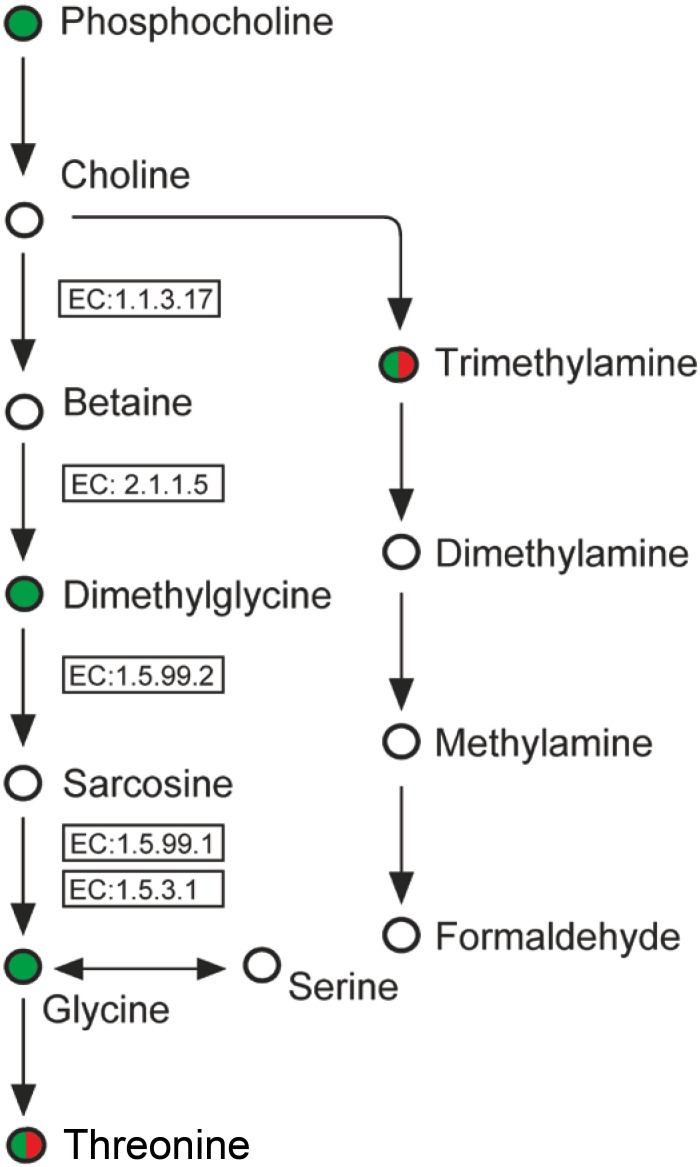
Schematic representation of metabolites related to choline. Identified metabolites from ^1^H NMR spectroscopy are colored, increased metabolites are shown in red, and decreased metabolites are in green. The left and right half circles represent changes in level of each component under acute and chronic Cu stress, respectively. Key enzymes in choline catabolism include EC 1.1.3.17, choline oxidase; EC 2.1.1.5, betaine-homocysteine methyltransferase; EC 1.5.99.2, demethylglycine dehydrogenase; EC 1.5.3.1, sarcosine oxidase; and EC 1.5.99.1, choline oxidase.

Though the quaternary ammonium compound choline, which is the major component of membrane lipids in eukaryotes [70], was not detected in the present study, its precursor o-phosphocholine was found to be decreasing in its content under Cu stress. The metabolic pathway related to choline is summarized in Fig. 5. Significant reductions to a similar extent were observed for phosphocholine, dimethylglycine and glycine in *S. fusiforme* under acute Cu stress, while a quite different metabolic pattern was observed for chronic stress, where trimethylamine showed the opposite behavior and increased 2.3-fold over the control. The function of trimethylamine in various maritime plants may be related to the common saline habitat, possibly in osmotic regulation or in the transport of ions across membranes [71], i.e. a kind of osmolytes, which principally are sugars, polyhydric alcohols, amino acids and their derivatives, and methylamines, and all are known to be protein stabilizers. This observation suggests trimethylamine be as the preferred osmoprotectant in *S. fusiforme* under chronic Cu stress, rather than dimethylglycine.

Another most common and widely distributed compatible osmolyte proline was only detected by GC-TOF MS (Fig. 2), but not NMR. In plants, proline is synthesized mainly from glutamate [72]. They showed similar behavior that without significant accumulations in the level to chronic Cu stress (Table 1 and Fig. 2). However, significant reductions were observed in *S. fusiforme* under acute Cu stress (Table 1 and Fig. 2). As intracellular proline levels are determined by biosynthesis, catabolism and transport between cells and different cellular compartments, we hypothesized that, dissimilar to other organisms, *S. fusiforme* may not use proline first as an osmoprotectant under Cu stress.

### Ascorbate: an important antioxidant component

The concentrations of all the other detected polyols, organic acids and sugars were decreased, ranging from 20% for myo-inositol to 49% for citrate (Table 2), especially ascorbate and lactate (90% and 79%, respectively), where the former is considered as the main antioxidants in many plants. Protective mechanisms in photosynthetic organisms do not only include ROS enzymes that reduce oxidative stress either with or without the aid of antioxidants but also antioxidants themselves [73].

In the brown algae *Scytosiphon lomentaria*, accumulation of ascorbate was detected in thalli from the Cu-enriched area [74]. In contrast, ascorbate content rapidly decreased and remained low in *Ulva compressa* (Chlorophyta) exposed to excess Cu [75]. Additionally, a low level of ascorbate was also observed in *U. compressa* collected in Cu-enriched environments, indicating that short-term responses induced by excess Cu were similar to long-term responses occurring in the level of ascorbate [76]. Rapid reduction (almost 90%) of ascorbate was caused by acute Cu exposure in *S. fusiforme* in this study. Though to a relatively smaller extent, the content of ascorbate in thalli decreased by approximately 58% after chronic Cu exposure as compared to the control.

Oxidative stress can be mitigated by the synthesis of antioxidant component ascorbate which directly reduces ROS [23]. It is of particular interest as an electron donor for •OH radicals and also as a substrate for ascorbate peroxidase (APX). An increase in ascorbate level was absent in *S. fusiforme*, similar to that in *U. compressa* that may be due to a direct oxidation of the newly synthesized ascorbate by Cu-induced ROS and/or to the activation of the antioxidant enzyme APX [77]. Cu^2+^ primarily triggers oxidase stress in the chloroplast, in which no active transport of ascorbate has been reported [78]. Therefore, reduced ascorbate was likely to be regenerated by the ascorbate/GSH cycle. As a result, rapid oxidation of ascorbate was provoked by acute Cu stress, which would abruptly generate of high levels of ROS over a short period that usually exceed the total antioxidant capacity of algae. Furthermore, it is reported that the decrease in ascorbate availability would as a result limit not only APX but also all peroxidase activity [78]. This to some extent explained the less activation in POD activity in thalli under acute Cu exposure in this study.

## Conclusions

In conclusion, we present metabolic profiles observed for *S. fusiforme* under both acute and chronic Cu exposures. In order to identify as many metabolites as possible, which is also the goal of untargeted metabolomics experiments, ^1^H NMR and GC-TOF MS were used complementally in the present study. Number of metabolites observed by MS platform was several times as that of NMR in this study, as NMR is generally considered to be less sensitive than GC-MS. These platforms would help expand our understanding of biological mechanisms related to environmental perturbations. Our results demonstrated different patterns of the marine brown algae *S. fusiforme* to acute and chronic Cu exposures in both physiological responses and regulation of metabolic pathways.

## References

[1] Kunhikrishnan A, Bolan NS, Naidu R, Kim WI (2013) Recycled water sources influence the bioavailability of copper to earthworms. J Hazard Mater 261: 784–792. 23122192 10.1016/j.jhazmat.2012.10.015

[2] Järup L (2003) Hazards of heavy metal contamination. Br Med Bull 68: 167–182. 14757716 10.1093/bmb/ldg032

[3] Pang SJ, Shan TF, Zhang ZH, Sun JZ (2008) Cultivation of the intertidal brown alga *Hizikia fusiformis* (Harvey) Okamura: mass production of zygote-derived seedlings under commercial cultivation conditions, a case study experience. Aquac Res 39: 1408–1415.

[4] Fang J, Wang KX, Tang JL, Wang M, Ren SJ, et al (2004) Copper, Lead, Zinc, Cadmium, Mercury, and Arsenic in Marine Products of Commerce from Zhejiang Coastal Area, China, May 1998. Bull Environ Contam Toxicol 73: 583–590. 15386182 10.1007/s00128-004-0468-z

[5] Contreras L, Mella D, Moenne A, Correa JA (2009) Differential responses to copper-induced oxidative stress in the marine macroalgae *Lessonia nigrescens* and *Scytosiphon lomentaria* (Phaeophyceae). Aquat Toxicol 94: 94–102. 19581008 10.1016/j.aquatox.2009.06.004

[6] Wu TM, Hsu YT, Sung MS, Hsu YT, Lee TM (2009) Expression of genes involved in redox homeostasis and antioxidant defense in a marine macroalga *Ulva fasciata* by excess copper. Aquat Toxicol 94: 275–285. 19665240 10.1016/j.aquatox.2009.07.010

[7] Okamoto OK, Pinto E, Latorre LR, Bechara EJH, Colepicolo P (2001) Antioxidant Modulation in Response to Metal-Induced Oxidative Stress in Algal Chloroplasts. Arch Environ Contam Toxicol 40: 18–24. 11116337 10.1007/s002440010144

[8] Dowling VA, Sheehan D (2006) Proteomics as a route to identification of toxicity targets in environmental toxicology. Proteomics 6: 5597–5604. 16972288 10.1002/pmic.200600274

[9] van Lipzig MM, Commandeur JN, de Kanter FJ, Damsten MC, Vermeulen NP, et al (2005) Bioactivation of Dibrominated Biphenyls by Cytochrome P450 Activity to Metabolites with Estrogenic Activity and Estrogen Sulfotransferase Inhibition Capacity. Chem Res Toxicol 18: 1691–1700. 16300378 10.1021/tx0501233

[10] Wu H, Wang WX (2011) Tissue-specific toxicological effects of cadmium in green mussel (*Perna viridis*): Nuclear magnetic resonance-based metabolomics study. Environ Toxicol Chem 30: 806–812. 21184531 10.1002/etc.446

[11] Zhang L, Liu X, You L, Zhou D, Wu H, et al (2011) Metabolic responses in gills of Manila clam *Ruditapes philippinarum* exposed to copper using NMR-based metabolomics. Mar Environ Res 72: 33–39. 21632102 10.1016/j.marenvres.2011.04.002

[12] Wu H, Liu X, Zhao J, Yu J, Pang Q, et al (2012) Toxicological effects of environmentally relevant lead and zinc in halophyte *Suaeda salsa* by NMR-based metabolomics. Ecotoxicol 21: 2363–2371. 10.1007/s10646-012-0992-2 22926641

[13] Wu H, Liu X, Zhao J, Yu J (2011) NMR-Based metabolomic investigations on the differential responses in adductor muscles from two pedigrees of Manila clam *Ruditapes philippinarum* to cadmium and zinc. Mar Drugs 9: 1566–1579. 22131959 10.3390/md9091566 PMC3225936

[14] Wu H, Liu X, Zhao J, Yu J (2012) Toxicological responses in halophyte *Suaeda salsa* to mercury under environmentally relevant salinity. Ecotoxicol Environ Saf 85: 64–71. 22947507 10.1016/j.ecoenv.2012.03.016

[15] Liu X, Zhang L, You L, Cong M, Zhao J, et al (2011) Toxicological responses to acute mercury exposure for three species of Manila clam *Ruditapes philippinarum* by NMR-based metabolomics. Environ Toxicol Pharmacol 31: 323–332. 21787701 10.1016/j.etap.2010.12.003

[16] Wu H, Zhang X, Li X, Li Z, Wu Y, et al (2005) Studies on the acute biochemical effects of La(NO_3_)_3_ using ^1^H NMR spectroscopy of urine combined with pattern recognition. J Inorg Biochem 99: 644–650. 15621299 10.1016/j.jinorgbio.2004.11.021

[17] Liu X, Yang C, Zhang L, Li L, Liu S, et al (2011) Metabolic profiling of cadmium-induced effects in one pioneer intertidal halophyte *Suaeda salsa* by NMR-based metabolomics. Ecotoxicol 20: 1422–1432. 10.1007/s10646-011-0699-9 21573875

[18] Farag MA, Porzel A, Wessjohann LA (2012) Comparative metabolite profiling and fingerprinting of medicinal licorice roots using a multiplex approach of GC–MS, LC–MS and 1D NMR techniques. Phytochem 76: 60–72. 10.1016/j.phytochem.2011.12.010 22336263

[19] Barding GA, Béni S, Fukao T, Bailey-Serres J, Larive CK (2012) Comparison of GC-MS and NMR for Metabolite Profiling of Rice Subjected to Submergence Stress. J Proteome Res 12: 898–909. 23205590 10.1021/pr300953k

[20] Dunn WB, Erban A, Weber R, Creek D, Brown M, et al (2013) Mass appeal: metabolite identification in mass spectrometry-focused untargeted metabolomics. Metabolomics 9: 44–66.

[21] Pan Z, Raftery D (2007) Comparing and combining NMR spectroscopy and mass spectrometry in metabolomics. Anal Bioanal Chem 387: 525–527. 16955259 10.1007/s00216-006-0687-8

[22] Barding GA, Fukao T, Beni S, Bailey-Serres J, Larive CK (2012) Differential metabolic regulation governed by the rice SUB1A gene during submergence stress and identification of alanylglycine by ^1^H NMR spectroscopy. J Proteome Res 11: 320–330. 22017194 10.1021/pr200919b

[23] Foyer CH, Noctor G (2011) Ascorbate and Glutathione: The Heart of the Redox Hub. Plant Physiol 155: 2–18. 21205630 10.1104/pp.110.167569 PMC3075780

[24] Bradford MM (1976) A rapid and sensitive method for the quantitation of microgram quantities of protein utilizing the principle of protein-dye binding. Anal Biochem 72: 248–254. 942051 10.1016/0003-2697(76)90527-3

[25] Zhang L, Liu X, You L, Zhou D, Wang Q, et al (2011) Benzo(a)pyrene-induced metabolic responses in Manila clam *Ruditapes philippinarum* by proton nuclear magnetic resonance (^1^H NMR) based metabolomics. Environ Toxicol Pharmacol 32: 218–225. 21843802 10.1016/j.etap.2011.05.006

[26] Wu H, Zhang X, Wang Q, Li L, Ji C (2013) A metabolomic investigation on arsenic-induced toxicological effects in the clam *Ruditapes philippinarum* under different salinities. Ecotoxicol Environ Safety: 90, 1–6. 23374855 10.1016/j.ecoenv.2012.02.022

[27] Wu H, Liu X, Zhao J, Yu J (2013) Regulation of Metabolites, Gene Expression, and Antioxidant Enzymes to Environmentally Relevant Lead and Zinc in the Halophyte *Suaeda salsa* . J Plant Growth Regul 32: 353–361.

[28] Parul VP, David MR, Mark RV, David LW (2004) Discrimination Models Using Variance-Stabilizing Transformation of Metabolomic NMR Data. OMICS: J Integr Biol 8: 118–130. 10.1089/1536231041388348 15268771

[29] Fan JH, Xie GZ, Wen SL (1996) The relativistic beaming model for active galactic nuclei. Astron Astrophys Suppl Ser 116: 409–415.

[30] Lisec J, Schauer N, Kopka J, Willmitzer L, Fernie AR (2006) Gas chromatography mass spectrometry-based metabolite profiling in plants. Nat Protoc 1: 387–396. 17406261 10.1038/nprot.2006.59

[31] Romanowska E (2002) Gas Exchange Functions in Heavy Metal Stressed Plants. In: Prasad MNV, Strzałka K editor. Physiology and Biochemistry of Metal Toxicity and Tolerance in Plants. Springer Netherlands, Berlin. pp. 257–285.

[32] Zeier J (2013) New insights into the regulation of plant immunity by amino acid metabolic pathways. Plant Cell Environ 36: 2085–2103. 23611692 10.1111/pce.12122

[33] Díaz J, Bernal A, Pomar F, Merino F (2001) Induction of shikimate dehydrogenase and peroxidase in pepper (*Capsicum annuum* L.) seedlings in response to copper stress and its relation to lignification. Plant Sci 161: 179–188.

[34] Richards TA, Dacks JB, Campbell SA, Blanchard JL, Foster PG, et al (2006) Evolutionary Origins of the Eukaryotic Shikimate Pathway: Gene Fusions, Horizontal Gene Transfer, and Endosymbiotic Replacements. Eukaryot Cell 5: 1517–1531. 16963634 10.1128/EC.00106-06 PMC1563581

[35] Pelletreau KN, Targett NM (2008) New Perspectives for Addressing Patterns of Secondary Metabolites in Marine Macroalgae. In: Amsler CD editor. Algal Chemical Ecology. Springer Berlin Heidelberg. pp. 121–146.

[36] Fernandez E, Galvan A (2008) Nitrate Assimilation in Chlamydomonas. Eukaryot Cell 7: 555–559. 18310352 10.1128/EC.00431-07 PMC2292633

[37] Campbell WH (1999) Nitrate Reductase Structure, Function and Regulation: Bridging the Gap between Biochemistry and Physiology. Annu. Rev. Plant Physiol. Plant Mol Biol 50: 277–303. 10.1146/annurev.arplant.50.1.277 15012211

[38] Harrison WG, Eppley RW, Renger EH (1977) Phytoplankton Nitrogen Metabolism, Nitrogen Budgets, and Observations on Copper Toxicity: Controlled Ecosystem Pollution Experiment. Bull Marine Sci 27: 44–57.

[39] Luna CM, Casano LM, Trippi VS (1997) Nitrate reductase is inhibited in leaves of *Triticum aestivum* treated with high levels of copper. Physiologia Plantarum 101: 103–108.

[40] Llorens N, Arola L, Bladé C, Mas A (2000) Effects of copper exposure upon nitrogen metabolism in tissue cultured *Vitis vinifera* . Plant Sci 160: 159–163. 11164588 10.1016/s0168-9452(00)00379-4

[41] Gouia H, Habib Ghorbal M, Meyer C (2000) Effects of cadmium on activity of nitrate reductase and on other enzymes of the nitrate assimilation pathway in bean. Plant Physiol Biochem 38: 629–638.

[42] Fontaine JX, Saladino F, Agrimonti C, Bedu M, Tercé-Laforgue T, et al (2006) Control of the Synthesis and Subcellular Targeting of the Two GDH Genes Products in Leaves and Stems of *Nicotiana plumbaginifolia* and *Arabidopsis thaliana* . Plant Cell Physiol 47: 410–418. 16418233 10.1093/pcp/pcj008

[43] Lam HM, Coschigano KT, Oliveira IC, Melo-Oliveira R, Coruzzi GM (1996) The molecular-genetics of nitrogen assimilation into amino acids in higher plants. Annu Rev Plant Physiol Plant Mol Biol 47: 569–593. 15012301 10.1146/annurev.arplant.47.1.569

[44] Burzyñski M, Buczek J (1997) The effect of Cu^2+^ on uptake and assimilation of ammonium by cucumber seedlings. Acta Physiol Plantarum 19: 3–8.

[45] Ritter A, Ubertini M, Romac S, Gaillard F, Delage L, et al (2010) Copper stress proteomics highlights local adaptation of two strains of the model brown alga *Ectocarpus siliculosus* . Proteomics 10: 2074–2088. 20373519 10.1002/pmic.200900004

[46] Flynn KJ, Dickson DMJ, Al-Amoudi OA (1989) The ratio of glutamine:glutamate in microalgae: a biomarker for N-status suitable for use at natural cell densities. J Plankton Res 11: 165–170.

[47] Zhu X, Zou D, Du H (2011) Physiological responses of *Hizikia fusiformis* to copper and cadmium exposure. Botanica Marina 54: 431.

[48] MacFarlane GR, Burchett MD (2001) Photosynthetic Pigments and Peroxidase Activity as Indicators of Heavy Metal Stress in the Grey Mangrove, *Avicennia marina* (Forsk.) Vierh Mar Pollut Bull. 42: 233–240. 10.1016/s0025-326x(00)00147-8 11381878

[49] Hassan HM, Scandalios JM (1990) Superoxide dismutases in aerobic organisms. In: Alscher RG, Cumming JR editor. Stress Responses in Plants: Adaptation and Acclimatation Mechanisms. Wiley-Liss, New York. pp. 178–199.

[50] Asada K, Takahashi M (1987) Production and scavenging of active oxygen in photosynthesis. In: Kyle DJ, Osmond CB, Arntzen CJ editor. Photoinhibition. Elsevier, Amsterdam. pp. 227–287.

[51] Wu TM, Lee TM (2008) Regulation of activity and gene expression of antioxidant enzymes in *Ulva fasciata* Delile (Ulvales, Chlorophyta) in response to excess copper. Phycologia 47: 346–360.

[52] Cakmak I (2000) Possible roles of zinc in protecting plant cells from damage by reactive oxygen species. New Phytol 146: 185–205. 10.1046/j.1469-8137.2000.00630.x 33862977

[53] Bischof K, Rautenberger R (2012) Seaweed Responses to Environmental Stress: Reactive Oxygen and Antioxidative Strategies. In: Wiencke C, Bischof K editor. Seaweed Biology. Springer Berlin Heidelberg. pp. 109–132.

[54] Wickens G (2001) Human and Animal Nutrition. In: Wickens GE editor. Economic Botany. Springer Netherlands. pp. 127–149.

[55] Iwamoto K, Shiraiwa Y (2005) Salt-Regulated Mannitol Metabolism in Algae. Mar Biotechnol 7: 407–415. 16088352 10.1007/s10126-005-0029-4

[56] Reed RH, Davison IR, Chudek JA, Foster R (1985) The osmotic role of mannitol in the Phaeophyta: an appraisal. Phycologia 24: 35–47.

[57] Overnell J (1975) The effect of heavy metals on photosynthesis and loss of cell potassium in two species of marine algae, *Dunaliella tertiolecta* and *Phaeodactylum tricornutum* . Mar Biol 29: 99–103.

[58] Sunda WG, Huntsman SA (1983) Effect of competitive interactions between manganese and copper on cellular manganese and growth in estuarine and oceanic species of the diatom *Thalassiosira* . Limnol Oceanography 28: 924–934.

[59] Smirnoff N, Cumbes QJ (1989) Hydroxyl radical scavenging activity of compatible solutes. Phytochemistry 28: 1057–1060.

[60] Shen B, Jensen RG, Bohnert HJ (1997) Increased Resistance to Oxidative Stress in Transgenic Plants by Targeting Mannitol Biosynthesis to Chloroplasts. Plant Physiol 113: 1177–1183. 9112772 10.1104/pp.113.4.1177 PMC158240

[61] Kawamitsu Y, Boyer JS (1999) Photosynthesis and carbon storage between tides in a brown alga, *Fucus vesiculosus* . Mar Biol 133: 361–369.

[62] Xie X, Wang G, Pan G, Sun J, Li J (2014) Development of oogonia of *Sargassum horneri* (Fucales, Heterokontophyta) and concomitant variations in PSII photosynthetic activities. Phycologia 53: 10–14.

[63] Xu J, Fan X, Zhang X, Xu D, Mou S (2012) Evidence of Coexistence of C_3_ and C_4_ Photosynthetic Pathways in a Green-Tide-Forming Alga, *Ulva prolifera* . PLoS ONE 7: e37438. 22616009 10.1371/journal.pone.0037438 PMC3353924

[64] Niu J, Hu H, Hu S, Wang G, Peng G, et al (2010) Analysis of expressed sequence tags from the *Ulva prolifera* (Chlorophyta). Chinese Journal of Oceanology and Limnology 28: 26–36.

[65] Gao S, Chen X, Yi Q, Wang G, Pan G, et al (2010) A strategy for the proliferation of *Ulva prolifera*, main causative species of green tides, with formation of sporangia by fragmentation. PLoS One 5: e8571. 20052408 10.1371/journal.pone.0008571 PMC2797376

[66] Doubnerová V, Ryšlavá H (2011) What can enzymes of C_4_ photosynthesis do for C_3_ plants under stress? Plant Sci 180: 575–583. 21421406 10.1016/j.plantsci.2010.12.005

[67] Nagahisa E, Kan-no N, Sato M, Sato Y (1994) Variations in D-aspartate content with season and part of *Hizikia fusiformis* . Fisheries Sci 60: 777–779.

[68] Yokoyama T, Amano M, Sekine M, Homma H, Tokuda M (2011) Immunohistochemical Localization of Endogenous D-Aspartate in the Marine Brown Alga *Sargassum fusiforme* . Biosci Biotechnol Biochem 75: 1481–1484. 21821953 10.1271/bbb.110184

[69] Funakoshi M, Sekine M, Katane M, Furuchi T, Yohda M, et al (2008) Cloning and functional characterization of *Arabidopsis thaliana* D-amino acid aminotransferase D-aspartate behavior during germination. FEBS J 275: 1188–1200. 18318836 10.1111/j.1742-4658.2008.06279.x

[70] Chen C, Li S, McKeever DR, Beattie GA (2013) The widespread plant-colonizing bacterial species *Pseudomonas syringae* detects and exploits an extracellular pool of choline in hosts. Plant J 75: 891–902. 23763788 10.1111/tpj.12262

[71] Smith TA (1971) The occurrence, metabolism and functions of amines in plants. Biological Rev 46: 201–241. 10.1111/j.1469-185x.1971.tb01182.x 4942955

[72] Szabados L, Savouré A (2010) Proline: a multifunctional amino acid. Trends Plant Sci 15: 89–97. 20036181 10.1016/j.tplants.2009.11.009

[73] Collén J, Davison IR (1999) Reactive oxygen metabolism in intertidal *Fucus* spp. (Phaeophyceae). J Phycol 35: 62–69.

[74] Contreras L, Moenne A, Correa JA (2005) Antioxidant responses in *Scytosiphon lomentaria* (Phaeophyceae) inhabiting copper-enriched coastal environments. J Phycol 41: 1184–1195.

[75] Mellado M, Contreras RA, González A, Dennett G, Moenne A (2012) Copper-induced synthesis of ascorbate, glutathione and phytochelatins in the marine alga *Ulva compressa* (Chlorophyta). Plant Physiol Biochem 51: 102–108. 22153245 10.1016/j.plaphy.2011.10.007

[76] Ratkevicius N, Correa JA, Moenne A (2003) Copper accumulation, synthesis of ascorbate and activation of ascorbate peroxidase in *Enteromorpha compressa* (L.) Grev. (Chlorophyta) from heavy metal-enriched environments in northern Chile. Plant Cell Environ 26: 1599–1608.

[77] Gonzalez A, Vera J, Castro J, Dennett G, Mellado M, et al (2010) Co-occurring increases of calcium and organellar reactive oxygen species determine differential activation of antioxidant and defense enzymes in *Ulva compressa* (Chlorophyta) exposed to copper excess. Plant Cell Environ 33: 1627–1640. 20444222 10.1111/j.1365-3040.2010.02169.x

[78] Pinto E, Sigaud-kutner TCS, Leitão MAS, Okamoto OK, Morse D, et al (2003) Heavy metal-induced oxidative stress in algae. J Phycol 39: 1008–1018.

